# Notes and Notices

**Published:** 1896-01

**Authors:** 


### NOTES AND NOTICES.

ELECTROLYSIS FOR THE SURGICAL TREATMENT OF STRICTURES.* By J. A. Fort, M. D., Professor of Anatomy in the École Pratique of the Paris Faculte de Medecine. It affords me great pleasure to have the honor of being allowed, through the kindness of your President, to present to you a new instrument which I have devised and called "electrolyser," for the surgical treatment of strictures by the "linear electrolysis" method.

It is a well-known fact that electrolysis has been discarded on account of the imperfect instruments which were used. My electrolyser has all the advantages of the urethrotome and none of its inconveniences. It looks like a small whip, of which the handle contains a metallic wire projecting from the end, which connects with the flexible part. This instrument, being first introduced into the urethra, is connected with the negative pole of a continuous-current battery, and the positive pole is connected near the affected part, on the front of the thigh or over the pubes; then the current is turned on.

The operation, which is almost painless, requires thirty seconds (on an average), with a current of a strength of at least ten milliamperes, as indicated by means of a galvanometer. The electrolyser remains perfectly cool during the operation. In nearly all cases there is no bleeding, or but very little. The urethra is made asceptic before and after the operation, in order to prevent fever. I never allow a sound to remain permanently in the urethra for any length of time after the operation.

Usually the wound resulting from electrolysis heals quickly, without any local treatment whatever, and often the patient can attend to business imme-

---

* Read before the Section in Genito-Urinary Surgery of the New York Academy of Medicine, Tuesday, November 12th, 1895.

diately after the operation.* In nearly all cases I pass a sound the third day after the operation, also the day after. I instruct the patient to pass a sound. No. 22 or No. 24 F., every month and every other month.

With the urethrotome, which cuts blindly, the surgeon cannot ascertain the degree of density of the tissue of a stricture. On the contrary, by means of electrolysis, which merely produces a molecular destruction of the stricture, although the instrument remains cool, I have been able to demonstrate that there are two classes of strictures—"soft and hard." Hard strictures are in the proportion of one against five soft ones.

The time required to perform the operation varies with the density of the stricture. Some strictures are so hard that they cannot be successfully operated upon by electrolysis.

If my American colleagues who are familiar with the French language are willing to refer to one of my books entitled *Traitement des rêtrécissements par l'elēctrolysis linéaire* (this book can be procured at the library of the Academy of Medicine), they may find it quite interesting, as it will enable them to understand the improvements which have gradually been introduced in the applications of electrolysis to surgery during the last fifteen years. They will also understand how I have applied electrolysis to the treatment of strictures of the urethra, uterus, rectum, and œsophagus.

Up to date I have performed in Europe a hundred and thirty-five operations on strictures of the œsophagus (recorded in my book), and with the exception of those which were caused by malignant growths of the wall of the œsophagus, all recovered.

It has been my good fortune to meet here some leading surgeons who are authorities in the treatment of strictures, and I am very grateful to them for their kindness in giving me the opportunity to demonstrate the advantages of my method in operating upon some of their patients.

THE HAHNEMANN CLUB OF PHILADELPHIA, PA., one of the oldest of these local organizations in the country, gave a free course of lectures last winter to the homœopathic medical profession of that city. This winter it has taken up a line of important questions for debate.

At the meeting at Dr. Pemberton Dudley's, it discussed the various "Hindrances to the Progress of Homœopathy."

At the entertainment of Dr. Bushrod W. James, it debated the "Present Dangers to Homœopathy," which continued at the meeting held at the residence of Dr. John E. James.

At the last meeting at Dr. Aug. Korndoerfer's, it took up the discussion of "Hahnemann's Rules for Investigating the Curative Properties of Drugs."

The following questions have not yet been debated: What do you understand by the term "pura," as applied to our drug provings, and what means would you suggest as efficient safeguards against the introduction of heteroge-

---

* When the wound does not heal I merely prescribe injections morning and evening, with one part of hydrozone to twenty parts of water.

neous symptoms in the prover's records? The value of the so-called idiosyncrasies manifested in drug action upon given individuals as guiding symptoms to the selection of the homœopathic remedy for a given case of disease?

The Club proposes to celebrate the birthday of Hahnemann, on April 10th, by a general meeting of homœopaths in Philadelphia.

DR. BUSHROD W. JAMES, President of the Hahnemann Club of Philadelphia, Pa., suggests to the American homœopathic profession the proper celebration of Hahnemann's birthday, Friday, April 10th, this year, by both the profession and the laity in general assemblage in every city and town of this country where Homœopathy has a footing.

It is the Hahnemann year, and should be greatly honored by all true adherents.

He further suggests that on that day a special effort be made to obtain subscriptions to complete the statue to Dr. Samuel Hahnemann, and that collections be made on this occasion by all to obtain the balance of the fund needed.

Let every local society in the country take action and do its utmost duty in this regard.

DR. J. H. ALLEN, of Logansport, Indiana, has been ill for a long time with traumatic spinal meningitis. Under the skillful treatment of Dr. J. R. Haynes he has recovered his health, and is now in active practice. In addition he is lecturing at Hering College, in Chicago, and in the Medical Mission School, also in Chicago.

THE NATIONAL ASSOCIATION OF PHYSICIANS AND SURGEONS will hold its annual meeting at Indianapolis, on January 15th. This Association has appointed a section on vaccination, and has invited papers on vaccination, both from those who are in favor of it and those who are opposed. For further information apply to the Secretary, D. R. C. Kelsey, 153 East Ohio Street, Indianapolis, Indiana.

ADVERTISING AXIOMS, by J. Walter Thompson, of New York:

The reward of the faithful advertiser is certain.

Man advertises and the people make purchases.

"Many men, many minds." Many ads. in many publications, many buyers.

The name and quality of good goods can be advertised so as to be "more lasting than brass."

Make your ads. speak the truth boldly, and the people will appreciate your frankness and respond.

"From nothing (in the leading publications) nothing comes." From *something*, however, results are sure to come.

"May his fame endure forever"—the advertiser who advertises with sensible copy that appeals to the sense of the people.

### FUN FOR DOCTORS.

No Trouble at All.—Lady—"I am sorry to trouble you, doctor, and make you come all this distance. But I was too wretched to get along without your aid."

Doctor—"Never mind, madam; never mind. I have a patient next door whom I had to see, anyway. Killing two birds, you know."—*Texas Siftings.*

A Hard Question.—Doctor—"My goodness! This won't do. You don't eat enough."

Sick Boy—"You don't want me to eat, do you?"

Doctor—"Indeed I do."

Sick Boy (angrily)—"Then why in th' name o' sense did you tell me to take a big dose o' cod-liver oil before every meal?"—*Street & Smith's Good News.*

Ready for More.—Railroad King—"What do you think I need, doctor, to set me up again?"

Doctor—"Well, I think a little iron will help you."

Railroad King—"Good! I gobbled up a whole railroad system last week." —*Puck.*

Dolly—"Have you heard how Sadie is getting along?"

Molly—"She's better. She's taking seven different patent medicines."

Dolly—"Mercy on us! And are they all doing her good?"

Molly—"No; only one is."

Dolly—"Then why doesn't she leave off the others?"

Molly—"She's afraid to. She doesn't know which one it is."

Physician (to hospital nurse)—"You will see, if you please, that the patient is given the medicine exactly as prescribed."

Sick Man (formerly from Boston, feebly)—"If you have no objection, doctor, I greatly prefer that the medicine should be given the patient. That is the correct form."

Successful Treatment.—Mrs. Hojack—"Dr. Kapsool makes a specialty of reducing fat people, but Mr. Keedick has been under his treatment for a year without the desired result."

Hojack—"I am not so sure about that, Keedick mortgaged his house yesterday to pay Kapsool's bill."—*Truth.*

Mrs. Malaprop Again.—A regular physician was summoned to attend a member of Mrs. Malaprop's family. He expressed some surprise at being called, knowing the lady in question to be an ardent follower of Homœopathy. "Yes," said Mrs. M., "I have changed my views; the homœopaths are good for infant-ry, but the regulars are best for adult-ry."

